# The Vacation Period

**Published:** 1896-07

**Authors:** 


### Editorial.

### THE VACATION PERIOD.

The opening of the midsummer months is a period looked forward to generally as one of recreation, and the dentist, with the hours of increasing toil behind him, anxiously anticipates the time when the last patient has been seen, the office closed, and the rest of brain and body has begun.

It is unnecessary to elaborate upon the wearing character of the dental profession. Its sedentary work, the constant nerve-straining efforts with tortured patients, the hours of labor in unnatural positions, the overcoming of difficulties unexpectedly met with in practice, the anxieties as to results, all combine to make the practice of dentistry one of the most laborious men or women can follow.

The other side of the picture has, however, much that is attractive, so much so that very few are willing to abandon it when once thoroughly established. While its compensation financially may be inferior to many other professions, it is sufficiently remunerative to warrant earnest and continued application, while its pleasant environments and freedom from liability to the constant exactions of patients make it a preferable choice to the practice of medicine.

It is, unfortunately, too true that the practice of dentistry is not adapted to all constitutions, and it is equally true that its in-door work has been and will continue to be attractive to many who should select an out-door occupation. The sedentary life of the dentist is not fitted to those who have inherited or acquired tendencies to lung complications, and yet these, mistakingly too often, are found in the colleges preparing to accept this as the work of their lives. If the preceptors of these young men would do their duty in the premises, the ambitious, but not overstrong, would be properly advised; but it is very apparent to those who have had much experience in teaching that this is too often neglected.

It is possible that it might be an error of judgment to advise a young person not to adopt a profession for the reason given, but it would certainly be almost a crime to suggest its selection unless its dangers to an already weakened constitution were fully portrayed, as well as the probable remedy. That it is possible to practise dentistry in the face of much inherited physical weakness has been too often in evidence to need any argument; but when this has been successfully accomplished, it has been through an intelligent comprehension of the difficulties and the possibilities of overcoming them by effective sanitary measures.

It is an axiom in the active life of the world "that to the young all things are possible;" but this is simply an overstatement of a truth, that with youth, energy, and strength the world is an open arena for all such to make a place. In this sense it is true of dentistry as of all other occupations. To accomplish this, health is the first object of interest and not money. Where the latter overrides everything then the naturally weak must go to the wall.

It is pitiable to see young men with bent shoulders, hollow chests, and attenuated forms struggling over hysterical patients, and hourly, daily, and weekly going through this life-destroying work without the apparent consciousness that there is a limit to human energy, and that when the curtain is rung down prematurely and the family left to fight, as best it may, life's battles, the vain regrets will not suffice as a compensation.

What, then, may be the remedy not only for those who rejoice in their strength, but the physically incapacitated for hard out-door labor? It is, in a word, to moderate the desire for gain.

The idea too generally prevails that an hour lost in the working hours of the day is just so many dollars sacrificed out of the family treasury, and that, therefore, if in full practice, the engagement with the first patient must begin early and the last end late. Is it possible that such a day can be satisfactory, even admitting that the physical organism can endure it? It is certainly within reason and experience to infer that a nervously racked person is incapable of operating with either justice to himself or patient, and he has no moral right to fail in his best efforts. The remedy is plain that, aside from shorter hours, there should be a relaxation of overstrain some time through the working periods, and, in addition, the nervous system should be thoroughly braced for the day's work by exercise in the open air in the early morning, with a short walk at the noon hour in the sun.

With the present facilities of taking exercise, combined with exhilarating pleasure on the bicycle, there can be no reasonable excuse for the ills of a sedentary life.

The writer has been often saddened by the observation of wrecked lives in the dental profession through a lack of attention to plain sanitary rules well understood, but entirely neglected. Those who stand in the position of teachers or leaders of thought cannot too frequently or too strongly instruct the energetic youth seeking to enter our ranks that devotion to their profession does not include self-sacrifice.

In addition to the daily recreation there should be added the summer vacation. Let the office rest, close the doors, and hie away to the free air and life of nature in her more perfect surroundings. The active worker during the busy months of the year has but a limited time for literary pursuits, or even to meet the intellectual demands of his profession, and, as a result, dentists are, as a rule, not as intelligent as they should be on the subjects vital to their best interests or to the progress made in their calling. With one or, better, two months of freedom, there will be a positive gain, and all should take this amount to give time for recuperation and opportunity for rest and the cultivation of the mind on professional and collateral topics.

The annual conventions should be attended. Very few appreciate the energizing influence these have upon the work of the year. What but this can be the inducement for men to travel hundreds of miles to attend a meeting, and nothing is able to draw their attention elsewhere. Certainly this incentive cannot be found in the scientific pabulum received, for this is often very meagre; but it is to be looked for in that contact with men engaged in the same occupation, the renewal of old friendships, and in that indefinable magnetism which comes with mingling in large bodies, filling and satisfying the nature as nothing else will or can. The life of a dentist without this seems to the writer poor indeed.

It is a subject for regret that so many absent themselves from these annual convocations who are the active spirits in their local organizations. This is a mistake, and should be remedied.

In July and August the two great national bodies—the Southern Dental Association and the American Dental Association—meet in separate localities. It is to be regretted that any dividing lines exist; but as these are not as yet closed up and one national body established, it should be the duty of those able to go, in both sections, to wend their way to these meetings. The young man may not find the first year satisfactory; but continue to go, and the value of professional intercourse will become apparent, and in the end the conclusion will be that a week with the national body is worth months of isolation. With renewed health and a brain rendered more active, the busy months of the rest of the year will go by with a feeling of thankfulness that a vacation was taken when most needed, and that the time was not wholly lost to the profession.

